# Resilience in advanced cancer patients who obtain a long-term response to immunotherapy or targeted therapy: an Ecological Momentary Assessment study

**DOI:** 10.1093/abm/kaaf042

**Published:** 2025-06-21

**Authors:** Laura C Zwanenburg, Eeske van Roekel, Karijn P M Suijkerbuijk, José J Koldenhof, Olga C J Schuurbiers-Siebers, Janneke van der Stap, Marije L van der Lee, Melanie P J Schellekens

**Affiliations:** Tilburg University School of Social and Behavioral Sciences, Center of Research on Psychological Disorders and Somatic Diseases, Department of Medical and Clinical Psychology, Tilburg, The Netherlands; Helen Dowling Institute, Centre for Psycho-Oncology, Scientific Research Department, Bilthoven, The Netherlands; Tilburg University School of Social and Behavioral Sciences, Center of Research on Psychological Disorders and Somatic Diseases, Department of Developmental Psychology, Tilburg, The Netherlands; Tilburg University School of Social and Behavioral Sciences, Tilburg Experience Sampling Center, Tilburg, The Netherlands; University Medical Centre in Utrecht, Department of Medical Oncology, Utrecht University, Utrecht, The Netherlands; University Medical Centre in Utrecht, Department of Medical Oncology, Utrecht University, Utrecht, The Netherlands; Radboud University Medical Centre, Department of Pulmonary Diseases, Nijmegen, The Netherlands; University Medical Centre in Utrecht, Department of Lung Diseases, Utrecht University, Utrecht, The Netherlands; Tilburg University School of Social and Behavioral Sciences, Center of Research on Psychological Disorders and Somatic Diseases, Department of Medical and Clinical Psychology, Tilburg, The Netherlands; Helen Dowling Institute, Centre for Psycho-Oncology, Scientific Research Department, Bilthoven, The Netherlands; Tilburg University School of Social and Behavioral Sciences, Center of Research on Psychological Disorders and Somatic Diseases, Department of Medical and Clinical Psychology, Tilburg, The Netherlands; Helen Dowling Institute, Centre for Psycho-Oncology, Scientific Research Department, Bilthoven, The Netherlands

**Keywords:** resilience, ecological momentary assessment, melanoma, lung cancer, immunotherapy, targeted therapy

## Abstract

**Background:**

Approximately half of advanced cancer patients with a long-term response to immuno- or targeted therapy (IT/TT) (ie, long-term responders (LTRs)) experience heightened distress due to persistent uncertainty.

**Purpose:**

We aimed to study to what extent supportive factors (ie, illness acceptance, tolerance of uncertainty, mindfulness, social support, optimism, emotion regulation variability, and positive affect in general and prior to a stressor) predict micro-level resilience in response to unpleasant daily life events.

**Methods:**

We conducted an observational cohort study with a baseline assessment of supportive factors, followed by Ecological Momentary Assessment with 8 assessments a day for 14 consecutive days. Resilience was operationalized as maintenance of low negative affect (NA) or a smaller increase in NA to an unpleasant event, as this suggests that partial recovery has already taken place. We used Dynamic Structural Equation Models to study supportive factors of resilience.

**Results:**

We included data from 61 patients with advanced melanoma or lung cancer with confirmed response to or long-term stable disease while on IT/TT. More unpleasant daily life events were associated with increases in NA. The multivariate model did not identify any supportive factors. Exploratory analysis using separate models tentatively indicated that LTRs with higher levels of illness acceptance, mindfulness, optimism, and general positive affect showed a smaller increase in NA in response to an unpleasant event (ie, more resilient response).

**Conclusions:**

Preliminary findings suggest that illness acceptance, mindfulness, optimism, and general positive affect are supportive factors of resilience in LTRs. Future research should include these factors at momentary level to enhance insight into the resilience process.

## Graphical Abstract

**Graphical Abstract:**
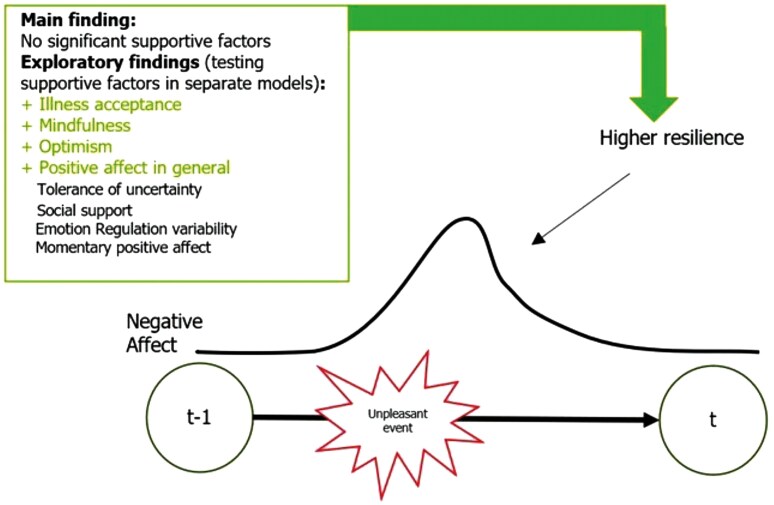

As a result of effective treatment with modern medical treatments such as immuno- or targeted therapy (IT/TT), the group of advanced (ie, metastatic) cancer patients who experience a prolonged survival is rapidly growing.^1,2^ Approximately half of the so-called long-term responders (ie, patients with advanced cancer who experience a prolonged survival, possibly for years, due to modern therapies such as IT/TT) (LTRs) report heightened levels of distress, because this durable response comes with many challenges.^3,4^ LTRs need to adjust to a new way of life in which death is a continuous threat, while being repeatedly confronted with uncertainties and other stressors, such as frequent check-ups and treatment side effects.^5^ Specific for LTRs is the ongoing uncertainty that shapes their experiences making them more prone to the development of mood or anxiety symptoms.^6^ For example, when planning a holiday they wonder whether they will still be here next summer.^4^

This ongoing uncertainty can make it difficult for LTRs to cope with unpleasant daily events. The ideal range of arousal for a person to function effectively in daily life is known as the window of tolerance.^7^ A major life event, such as getting an advanced cancer diagnosis, narrows one’s window of tolerance, increasing sensitivity to stress in everyday life. Previous research confirmed this theory, showing that although cancer patients report a similar number of stressful events in daily life as healthy controls, cancer patients tend to respond more sensitively to these stressors.^8^

## Resilience

The extent to which LTRs adaptively cope with stressors is defined as resilience. Resilience refers to a dynamic process of adaptation and is defined as the maintenance or quick recovery of mental health in response to adversity.^9^ Hence, resilience is not a trait, but something that is more or less present after deploying certain resources or skills in response to a stressor.^10^ From this point of view, resilience can be improved by empowering LTRs to recognize and employ these resources and abilities.

To improve our understanding of which resources or abilities (ie, supportive factors) contribute to resilience, the current research aims to study resilience in the ebb and flow of LTRs daily lives. Using Ecological Momentary Assessment (EMA), a structured diary technique to assess subjects in their daily living environment, we can investigate changes in people’s emotional reactions to stressors.^11^ These repeated in-the-moment assessments are ideally suited to gain insight into the daily life dynamics of resilience and its supportive factors.^12^

## Domains of positive functioning

A framework that provides insight into possible supportive factors of resilience is the empirically derived framework of the Five Domains of Positive Functioning (DPF-5).^13^ This framework, based on positive psychology research, distinguishes 5 partly overlapping domains that cover different aspects of how individuals achieve well-being: Attention and Awareness, Comprehension and Coping, Emotions, Goals and Habits, and Virtues and Relationships (see Electronic Supplementary Material 1).

The first domain, ‘Attention and awareness’, entails the conscious regulation of attention toward particular aspects of information.^13^ For example, *mindfulness* strategies encourage individuals to observe their experiences with open awareness and without judgment. This practice enables them to take a step back and consciously choose how they want to respond to stressful events.^14^ A recent scoping review highlights LTRs consider it helpful to concentrate on the present moment when dealing with the ongoing uncertainty.^15^ As a result, *mindfulness* can aid in building well-being and resilience.

The second domain ‘Comprehension and coping’ includes one’s mindset, expectancies, attribution, and reappraisal^13^ and covers promising supportive factors of resilience, such as *optimism*, *acceptance*, *tolerance of uncertainty* and the ability to flexibly engage in different *emotion regulation* strategies. Regarding *optimism*, expectations about the future are shaped by confidence in achieving a goal. Optimists perceive their desired goals as attainable, which leads them to face adversities proactively.^16^ Cancer patients who have positive thoughts about their treatment outcomes tend to emotionally endure treatment better, resulting in a higher quality of life and lower psychological distress.^17^ LTRs strive to maintain optimism regarding, for example, future treatment options. They hope that cancer could become a manageable chronic condition or even be cured.^15^ Concerning acceptance, having an *accepting* attitude towards one’s illness means acknowledging one is ill and simultaneously perceiving the ability to live with and master the consequences of their disease.^18,19^ A meta-analysis revealed that acceptance of cancer was indeed associated with less general distress, cancer-specific distress, depressive symptoms, and anxiety symptoms.^20^ To come to terms with their new reality, LTRs are trying to accept the burden of the disease and treatment and face the possibility of death.^15^ Regarding *tolerance of uncertainty,* it can be defined as a set of positive and negative thoughts, feelings, and actions triggered by realizing what we don’t know about particular aspects of the world. When a person has a high degree of uncertainty tolerance, negative events are perceived as less threatening. It has been associated with emotional wellbeing and less avoidance behavior (eg, suppressing one’s emotions about an upcoming medical check-up).^21^ Accepting the limited control inherent to their situation helps LTRs cope with uncertainty.^15^ Concerning emotion regulation, it can protect people from psychological distress. Instead of the traditional categorization in adaptive (eg, putting things in perspective) and maladaptive (eg, catastrophizing) strategies, research has acknowledged that the adaptiveness of emotion regulation strategies depends on the situational demands, since not every emotion regulation strategy is suitable for every situation.^22^ The ability to flexibly use different strategies depending on the demand of the situation is referred to as *emotion regulation variability* and is expected to shield people from distress^22^ and play a role in resilient functioning.

The third domain, ‘Emotions’, includes the experience of *positive affect* (PA).^13^ Positive emotions facilitate the development and strengthening of one’s abilities and resources, such as physical strength, social bonds, creativity, and new cognitive skills.^23^ These resources can be drawn upon in subsequent moments of adversity, contributing to resilience.^24^

Regarding the fourth domain “Goals and Habits” no specific factors were selected for the current study.^13^ To minimize participant burden, we limited the number of baseline questionnaires to a maximum of 5 . Consequently, we did not include a potential supportive factor for this category, as we did not find a good way of assessing the process of goal adjustment.

The fifth and last domain, “Virtues and Relationships” includes social, organizational, and romantic relationships.^13^  *Social support*, by one’s partner, family, or friends, is an often-mentioned contributor to resilience in cancer literature.^25^ Studies show that the experience of feeling understood by others stimulates connectivity in brain regions associated with cognitive control, self-reflection, stress adaptation, and emotion regulation^26^ and, as such, contributes to resilience. For LTRs, feeling supported and understood by their close others can prevent feelings of guilt or shame due to their inability to, for example, perform daily tasks.^15^

Supported by the DPF-5,^13^ the aforementioned research on the psycho-social well-being of cancer patients and their experiences reveals that promising supportive factors contributing to resilience include illness acceptance, tolerance of uncertainty, mindfulness, perceived social support, optimism, emotion regulation variability, and positive affect. These factors seem to fit the unique situation of LTRs and might protect LTRs from developing severe distress and psychopathology.

## Aim

**Figure 1.**
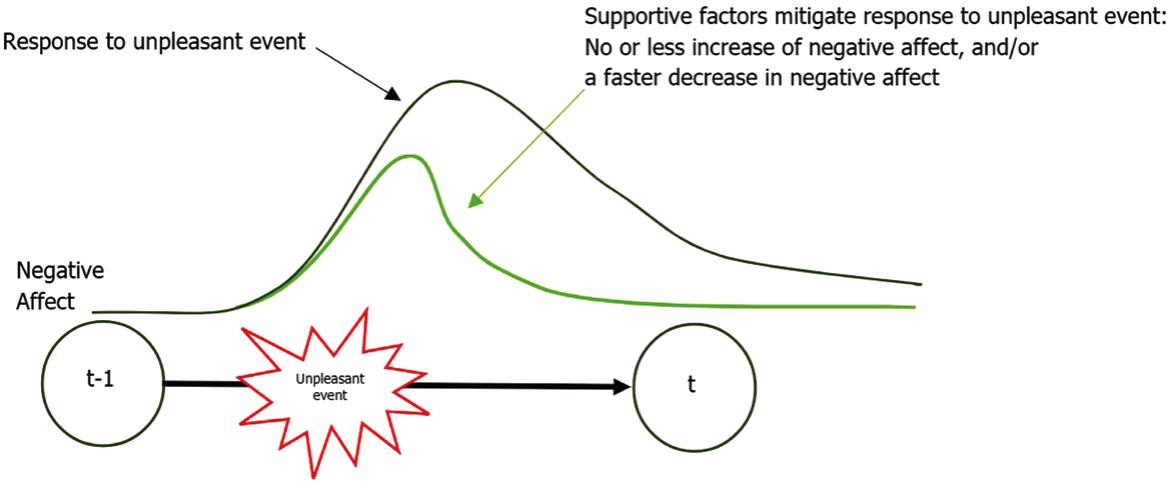
Depiction of hypothesis 2. *Note.* Figure shows how supportive factors are hypothesized to mitigate the response to an unpleasant event.

This study aims to identify supportive factors that foster resilience in advanced cancer patients obtaining a long-term response to IT/TT using EMA. We formulated the following research questions: 1. How do LTRs react to unpleasant daily life events? 2. Which supportive factors are predictors of resilience in LTRs? 3a. To what extent does the experience of PA in general predict resilience in LTRs? 3b. To what extent does the experience of PA before a stressor predict resilience in LTRs? We hypothesize that (H1) as the unpleasantness of an event increases, so does the level of negative affect. Higher levels of (H2) illness acceptance, tolerance of uncertainty, mindfulness, perceived social support, optimism, and emotion regulation variability are expected to mitigate this increase in negative affect following an unpleasant event (see Figure 1). Lastly, we presume that (H3a) general and (H3b) momentary PA alleviate this increase in negative affect following an unpleasant event.

## Methods

### Design

We conducted an observational cohort study gathering intensive longitudinal data with EMA over a period of 14 days to study resilience from a dynamic perspective. The study was pre-registered at the Open science Framework (see: https://osf.io/qpgda/) and approved by the Medical Ethics Committee Brabant, the Netherlands: NL78416.028.21.

### Participants

Participants needed to be diagnosed with advanced (ie, distantly metastatic or irresectable) melanoma or lung cancer. When studying long-term response to IT/TT among advanced cancer patients it is common to focus on lung cancer and melanoma as a relatively large group of patients respond well to IT/TT.^1,2^ Additionally, at least 2 scans were required to show a confirmed response to IT/TT. That is, a Response Evaluation Criteria in Solid Tumors (RECIST) score of at least a partial response (ie, a reduction of at least 30% in the sum of the longest diameters of target lesions, compared to the baseline sum of the longest diameters of the tumor) or more than one year of stable disease (ie, no sufficient shrinkage to qualify for partial response or sufficient increase to qualify for progressive disease, using the smallest sum of the longest diameters of the tumor since treatment began).^27^ Finally, participants needed to be at least 18 years of age, had a sufficient use and understanding of Dutch Language, and access to smartphone and internet.

### Procedures

Participants were recruited with continuous enrollment at University Medical Centre Utrecht, Radboud University Medical Centre, and the mental healthcare centre Helen Dowling Institute in the Netherlands. Healthcare professionals identified eligible patients and, in case of interest, asked permission to provide the researchers with contact details. A researcher contacted eligible patients to explain study procedures in more detail.

After providing informed consent, the researcher informed participants about EMA by downloading the smartphone application (Ethica software) on the participant’s phone and discussing the manual step by step.

One week after completing the online baseline questionnaires, participants started with the EMA questionnaires for 14 consecutive days. They received notifications every 105 minutes at 8 fixed time points a day to fill in the EMA items. We measured NA, the extent to which events were experienced as unpleasant, and PA at each time point. Emotion regulation strategy use was measured in the last EMA notification of the day. To prevent notifications from being missed, participants could choose notifications to start either at 8 a.m. or 9 a.m. Questionnaires remained available for 45 minutes after the notification. Researchers were available for questions on weekdays via telephone or e-mail. Researchers monitored the progress and contacted participants in case of repeated missing data to see if they could help and stimulate participants to complete as many assessments as possible. To thank participants for their participation, participants received a short, personalized feedback report of their daily life experiences, mood, and emotion regulation strategies (see Electronic Supplemental Material 2).

## Measures

### Baseline measures

**Illness acceptance.** An example item of the 6-item acceptance subscale (ICQ-A) of the Illness Cognitions Questionnaire (ICQ) is “I can handle the problems related to my illness” and could be answered on a 4-point Likert scale varying from 1 = “not at all” to 4 = ‘completely’. The ICQ-A has been shown to be valid and reliable.^28^ In the current study, internal consistency (α = 0.91) was excellent.

**Tolerance of uncertainty.** An example item of the 12-item Intolerance of Uncertainty Scale – short form (IUS-12) is ´Unforeseen events upset me greatly’ and was rated on a 5-point Likert scale varying from 1 = “completely disagree” to 5 = ‘totally agree’.^29,30^ Scores were reversed so that higher scores reflected higher tolerance of uncertainty. The IUS-12 shows good psychometric properties.^31^ In the current study, internal consistency (α = 0.87) was good.

**Mindfulness.** An example item of the 14-item Freiburg Mindfulness Inventory (FMI) is “I am open to the experience of the present moment” and was rated on a 4-point Likert scale varying from 1 = “rarely” to 4 = ‘almost always’.^32,33^ The FMI shows good psychometric qualities.^32^ In the current study, internal consistency (α = .88) was good.

**Social support.** An example item of the Multidimensional Scale of Perceived Social Support (MSPSS) is “My family really tries to help me” and was rated on a 7-point Likert scale varying from 1 = “completely disagree” to 7 = ‘totally agree’.^34^ The Dutch version of the MSPSS showed good psychometric qualities.^35^ In the current study, the MSPSS showed an excellent internal consistency (*α* 0.91).

**Optimism.** An example item of the 10-item (of which 4 filler items) Life Orientation Test-Revised (LOT-R) is “In uncertain times, I usually expect the best” and was answered on a 5-point Likert scale varying from 0 = “completely disagree” to 4 = ‘totally agree’.^36^ The Dutch LOT-R showed good psychometric qualities,^36^ with good internal consistency (α = 0.80) in the present study.

### EMA measures

Participants received 8 questionnaires per day between 08:00 a.m. and 20:15 p.m. or between 09:00 a.m. and 21:15 p.m.: 1 in the morning (11 items), 6 during the day (10 items), and 1 in the evening (28 items). A detailed overview of all EMA items is displayed in the pre-registration and supplemental materials. All EMA items were selected based on previous EMA studies among cancer patients and healthy adolescents by members of the research team and the EMA item repository,^37^ and showed adequate variability within individuals in general.^38,39^

**Negative affect.** At each EMA notification (8 times per day) 3 negative (ie, “I feel worried,” “I feel frustrated,” “I feel gloomy”) mood adjectives were assessed on a visual analogue scale (0 = “not at all”—100 = ‘very much’). NA refers to the mean score of the 3 mood items, showing good internal consistency (α = .83) in the present sample.

**Unpleasantness of events.** At each EMA notification (8 times per day) participants were asked to think about the most important event that happened since the previous notification. Subsequently, participants rated whether the event was perceived as pleasant or unpleasant on a 7-point bipolar Likert scale (-3 = “very unpleasant,” 0 = “neutral,” 3 = “very pleasant”). Following previous EMA studies,^40^ we recoded the negatively rated events so that a high score on this variable represented higher unpleasantness of events. As we were only interested in unpleasant events, the positive scores representing pleasant events were recoded as missing variables, and neutral events were included as the reference score (0).

**Resilience.** To study resilience from a dynamic perspective, resilience was operationalized in terms of daily life affect in response to an unpleasant event. NA is expected to increase in response to an unpleasant event, which is called reactivity. Higher levels of unpleasantness are expected to predict greater reactivity. The decrease of NA after the initial increase is called recovery (Electronic Supplementary Material 2). Following previous EMA research, resilience was operationalized as maintenance of NA (ie, no increase of NA from t-1 to t) or a smaller increase in NA to an unpleasant event compared to participants with less supportive factors available, as this suggests that partial recovery has already taken place.^41,42^

**Positive affect.** To measure PA, 2 positive (ie, “I feel satisfied,” ‘I feel cheerful’) mood adjectives were assessed on a visual analogue scale (0–100) at each notification, 8 times per day. Momentary PA refers to the mean score of the 2 mood items, showing excellent internal consistency (*α* = 0.95). General PA refers to the person-mean of PA across the EMA period.

**Emotion regulation variability.** At the last notification of the day (once per day) 10 items assessed emotion regulation strategies participants used in response to the most unpleasant event of the day (ie, sharing with others, distracting, acceptance, social support, having influence, pondering, trying to control one’s emotions, re-appraisal, expressing emotions, suppressing emotions) on a visual analogue scale (ranging from 0 = “not at all” to 100 = “very much”). Emotion regulation variability refers to choosing different strategies from a set of options at any given time, aiming to find the best one or prioritizing certain strategies based on the situational demands.^22^ For each individual, we calculated the standard deviation across all emotion regulation strategies at each occasion. Then, the standard deviations obtained at all occasions were averaged to compute the mean between-strategy variability at the participant level (ie, emotion regulation variability).^43^

## Statistical analysis

### Preregistered analysis plan

Data was prepared and descriptives and correlations were calculated using R software (version 2023.06.2 + 561). We used multi-level vector autoregressive models (MLVAR(1)) in *Mp*lus software (Version 8.8, 2022) to analyze data with Dynamic Structural Equation Models (DSEM). DSEM accounts for the hierarchical structure of EMA data, in which multiple observations (level 1) are nested within individuals (level 2) and prevents Type-1 errors. DSEM allows for to decomposition of variance on 2 levels: within-person (ie, variation from notification to notification) and between-person (ie, differences between individuals).^44^

Participants were included in the analysis if they completed more than one-third of the assessments (ie, > 37 assessments). A specific strength of DSEM in *Mp*lus is that it can handle missing values. Using the “tinterval” option^44,45^ with a tinterval of 120 minutes (ie, average time between assessments), we accounted for unequal spacing between assessments. Model settings were pre-registered at the Open Science Framework.

### Dynamic structural equation models

To study supportive factors of resilience, we specified 4 models (See Electronic Supplementary Material 3). To investigate to what extent LTRs show resilience in response to unpleasant daily life events, we examined whether unpleasantness of an event at t predicted changes in NA at t (NA_(t)_), while controlling for NA at the previous time point (NA_(t-1)_) (RQ1). Please note that the unpleasantness of an event was measured at the current time-point (t), but represented the unpleasantness of the most important event since the previous assessment. All within-person predictors were person-mean centered. The effects were specified as random (ie, allowing for individual differences in the lagged effects). On the between-person level, associations between the stable means (ie, random intercepts) of unpleasantness of an event, NA, and random slopes were estimated.

**Figure 2.**
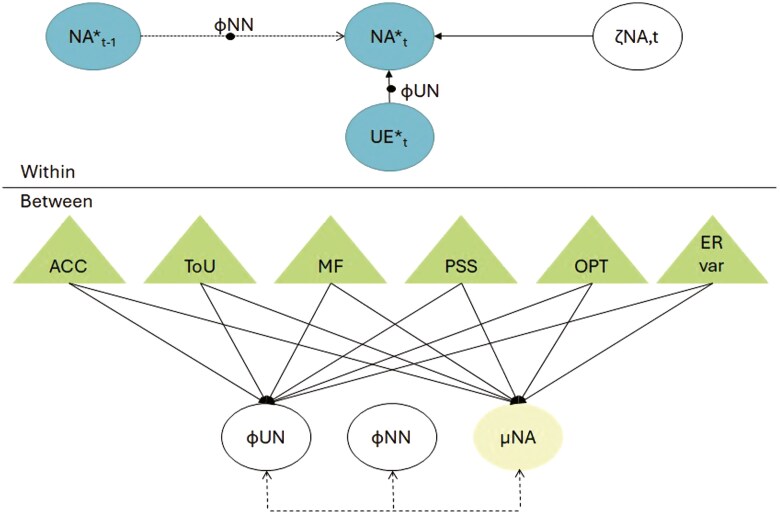
Model specification RQ 2. *Note*. NA = negative affect, UE = unpleasantness of event, ACC = illness acceptance, ToU = tolerance of uncertainty, MF = mindfulness, PSS = perceived social support, OPT = optimism, ER var = Emotion Regulation variability. Variables are decomposed in between-person part (μNA = individuals mean, in blue), and within-person part (NA*_t_ = within-person centered score of negative affect, UE*_t_ = within-person centered score of unpleasantness of event, in orange). Top panel: On the within-person level, NA predicts itself over time and unpleasantness of the event predicts NA on the same time point (ϕNN; ϕ = regression parameter, *ζ* = innovation). Bottom panel: On the between-person level, these random effects and individual means are correlated and the baseline predictors (ie, ACC, ToU, MF, PSS, OPT, and ER variability) are added as predictors of the intercept of NA and the slope of UE*_t_ on NA*_t_ (ϕUN) (in green).

To examine which supportive factors predict resilience in LTRs (RQ2), we tested to what extent between-person factors (ie, Illness acceptance, tolerance of uncertainty, mindfulness, perceived social support, optimism, and emotion regulation variability) moderated the association between unpleasantness of an event and changes in NA. The 5 between-person factors were entered simultaneously into the first model as moderators of the association between the unpleasantness of an event and NA (Figure 2). In addition, and deviating from the pre-registration, the between-person factors were also analyzed in separate models.

To investigate whether the experience of general PA predicted resilience (RQ3a), we examined the moderating role of average PA at the between-person level on the association between unpleasantness of an event and changes in NA. Next, we studied whether momentary PA (ie, on the within-person level) moderated the association between unpleasantness of an event and changes in NA (RQ 3b), by examining the moderating effect of momentary PA_t-1_ on the association between unpleasantness of an event and changes in NA.

### Power

We calculated the sample size for testing a selection of 5 predictors using G*Power. When testing 5 predictors (ie, supportive factors) in multi-level regression analysis and considering a 20% dropout to detect an effect size of.25 with 80% power and an alpha of ≤0.05, we needed to recruit 70 participants. This argumentation was also included in the pre-registration. However, while writing the manuscript, the authors realized that this reasoning is not correct for the chosen analysis method. According to guidelines on the number of subjects and assessments needed for multi-level analysis using dynamic structural equation modeling, we needed to recruit at least 50 participants using a model with cross-level interactions, since we planned to include 112 EMA assessments per participant.^46^

## Results

### Sample

Between January 2022 and January 2023, 72 patients were enrolled, of whom 4 dropped out after the EMA instructions because of the expected burden of EMA, and 5 dropped out during EMA due to illness progression. All participants completed more than one-third of the EMA notifications (min. = 64; max. = 121). Due to missing baseline questionnaires (*n* = 1) and lack of variation in answers on the EMA questionnaires (*n* = 1), 2 additional participants were excluded from analysis. Sufficient variation in data is essential for accurately estimating relationships between variables in DSEM. Without enough variation, the model cannot distinguish between different levels of the variables, leading to unreliable or biased parameter estimates. Eventually, the 61 participants who were included were aged between 24 and 79 years (*M* = 58.26; *SD* = 11.62). Most participants were treated with immunotherapy (*n* = 39). Time since diagnosis varied from 8 to 120 months (*M* = 43.41; *SD* = 24.40). Table 1 presents participants’ sociodemographic and clinical characteristics. Treatment details can be found in Electronic Supplementary Material 4.

**Table 1:** *|* Sociodemographic and clinical characteristics of the 61 participants.

	*n*	(%)
Age, mean (SD)	58.26	11.62
Gender		
Men	28	45.90
Women	33	54.10
Educational level		
Practical	6	9.84
Intermediate	20	32.79
Theoretical	35	57.38
Working status		
Employed/student	16	26.23
Unemployed	5	8.20
Disabled	17	27.87
Sick	5	8.20
Retired	16	26.23
Volunteering	2	3.28
Relationship status		
Single	4	6.56
Married/living together	53	86.88
Divorced	3	4.92
Widow	1	1.64
Cancer type		
Lung cancer	34	55.74
Melanoma	27	44.26
Time since diagnosis, M (SD)		
Diagnosis in months	43.41	24.40
Start current treatment in months	34.66	21.37
Best response in months	27.87	22.56
Treatment		
Immunotherapy	39	63.93
Targeted therapy	22	36.10

*Note*: SD = standard deviation.

### Descriptives and correlations

Participants completed a total of 6085 EMA notifications. Mean number of completed notifications was 99.75/112 (89.06%; *SD* = 10.44). In total, participants experienced 767 unpleasant events with an average of 13 unpleasant events per participant. They experienced an average of 0.90 unpleasant events per day (with a person-based mean varying between 0.00 to 3.29 unpleasant events per day). See Electronic Supplementary Material 5 for descriptive statistics.

### Dynamic structural equation models

All models converged, indicated by the Potential Scale Reduction (PSR) values < 1.071 for all parameters. Trace plots suggested successful convergence for all parameters. See Table 2 for estimates, standardized estimates, *P*-values, and 95% confidence intervals of all models.

**Table 2:** | Model results of preregistered dynamic structure equation models (ML-VAR) examining associations between unpleasantness of events and negative affect and the moderating effect of supportive factors.

	*B*	beta	*p*	95% CI
Model 1				
Within-person				
** UE**_**(t)**_ **→ NA**_**(t)**_ **(*ΦUN*)**	**3.791**	**0.293**	**0.000**	**[0.264; 0.320]**
** NA** _**(t—1)**_ **→ NA**_**(t)**_ **(*ΦNN*)**	**0.463**	**0.463**	**0.000**	**[0.434; 0.491]**
Model 2 (all between-person predictors simultaneously)				
Within-person				
** UE**_**(t)**_ **→ NA**_**(t)**_ **(*ΦUN*)**	**3.794**	**0.293**	**0.000**	**[0.265; 0.320]**
** NA** _**(t—1)**_ **→ NA**_**(t)**_ **(*ΦNN*)**	**0.466**	**0.466**	**0.000**	**[0.434; 0.495]**
Between-person				
Illness Acceptance	−0.206	−0.149	0.248	[-0.364; 0.094]
Tolerance of uncertainty	0.032	0.042	0.664	[-0.152; 0.226]
Mindfulness	−0.029	-0.036	0.772	[-0.290; 0.203]
Social Support	−0.094	-0.017	0.856	[-0.184; 0.151]
Optimism	−0.242	-0.208	0.060	[-0.404; 0.005]
ER variability	0.037	0.045	0.602	[-0.129; 0.222]
Model 2 (illness acceptance)				
Within-person				
** UE**_**(t)**_ **→ NA**_**(t)**_ **(*ΦUN*)**	**3.820**	**0.294**	**0.000**	**[0.266; 0.320]**
** NA** _**(t—1)**_ **→ NA**_**(t)**_ **(*ΦNN*)**	**0.467**	**0.466**	**0.000**	**[0.439; 0.495]**
Between-person				
** Illness acceptance**	**−0.311**	**−0.230**	**0.016**	**[−0.399; −0.039]**
Model 2 (Tolerance of Uncertainty)				
Within-person				
** UE**_**(t)**_ **→ NA**_**(t)**_ **(*ΦUN*)**	**3.821**	**0.294**	**0.000**	**[0.266; 0.320]**
** NA** _**(t—1)**_ **→ NA**_**(t)**_ **(*ΦNN*)**	**0.465**	**0.464**	**0.000**	**[0.437; 0.493]**
Between-person				
Tolerance of uncertainty	−0.055	−0.049	0.544	[−0.237; 0.124]
Model 2 (Mindfulness)				
Within-person				
** UE**_**(t)**_ **→ NA**_**(t)**_ **(*ΦUN*)**	**3.812**	**0.293**	**0.000**	**[0.266; 0.320]**
** NA** _**(t—1)**_ **→ NA**_**(t)**_ **(*ΦNN*)**	**0.466**	**0.466**	**0.000**	**[0.438; 0.494]**
Between-person				
** Mindfulness**	**−0.184**	**−0.237**	**0.010**	**[−0.402; −0.054]**
Model 2 (Social Support)				
Within−person				
** UE**_**(t)**_ **→ NA**_**(t)**_ **(*ΦUN*)**	**3.834**	**0.294**	**0.000**	**[0.267; 0.321]**
** NA** _**(t—1)**_ **→ NA**_**(t)**_ **(*ΦNN*)**	**0.464**	**0.463**	**0.000**	**[0.436; 0.492]**
Between−person				
Social Support	−0.476	−0.075	0.414	[−0.241; 0.102]
Model 2 (Optimism)				
Within-person				
** UE**_**(t)**_ **→ NA**_**(t)**_ **(*ΦUN*)**	**3.830**	**0.294**	**0.000**	**[0.267; 0.321]**
** NA** _**(t—1)**_ **→ NA**_**(t)**_ **(*ΦNN*)**	**0.467**	**0.466**	**0.000**	**[0.440; 0.495]**
	**B**	**beta**	***p***	**95% CI**
Between-person				
** Optimism**	**−0.316**	**−0.283**	**0.002**	**[−0.449; −0.098]**
Model 2 (ER variability)				
Within-person				
** UE**_**(t)**_ **→ NA**_**(t)**_ **(*ΦUN*)**	**3.819**	**0.293**	**0.000**	**[0.265; 0.320]**
** NA** _**(t—1)**_ **→ NA**_**(t)**_ **(*ΦNN*)**	**0.463**	**0.463**	**0.000**	**[0.436; 0.492]**
Between-person				
ER variability	0.011	0.013	0.872	[−0.160; 0.193]
Model 3 (PA mean)				
Within-person				
** UE**_**(t)**_ **→ NA**_**(t)**_ **(*ΦUN*)**	**3.835**	**0.295**	**0.000**	**[0.267; 0.322]**
** NA** _**(t—1)**_ **→ NA**_**(t)**_ **(*ΦNN*)**	**0.468**	**0.468**	**0.000**	**[0.439; 0.497]**
Between-person				
** PA mean**	**−0.120**	**−0.320**	**0.000**	**[−0.488; −0.134]**
Model 3 (PA_**(t—1)**_)				
Within-person				
** UE**_**(t)**_ **→ NA**_**(t)**_ **(*ΦUN*)**	**4.153**	**0.270**	**0.000**	**[0.243; 0.296]**
** NA** _**(t—1)**_ **→ NA**_**(t)**_ **(*ΦNN*)**	**1.708**	**1.708**	**0.000**	**[1.192; 2.281]**
UExPA_**(t—1)**_ → NA_(t)_	0.024	0.028	0.150	[−0.011; 0.063]

*Note*: UE = unpleasantness of an event, NA = negative affect, B = unstandardized estimates; beta = standardized estimates for fixed within- and between-person effects using the STDYX Standardization (within-level standardized estimates averaged over clusters) in Mplus. The between-person coefficients are indicating differences in slopes between individuals. *p* = Bayesian equivalent to 2-sided p-values. 95% CI = 95% credibility interval of standardized values. Significant associations are indicated in bold.

#### Research aim 1

The first model tested whether the unpleasantness of an event predicted changes in NA (ie, reactivity). Unpleasantness of an event significantly and positively predicted changes in LTRs’ NA (*β*=.29, *P* =.00). This means that when an individual’s score on unpleasant events increased by one SD, NA increased with 0.29 (person-specific) standard deviations. Hence, the more unpleasant the daily life event, the bigger the increase in NA.

#### Research aim 2

The second model tested whether Illness Acceptance, Tolerance of Uncertainty, Mindfulness, Social Support, Optimism, and ER variability protected LTRs from the effect of unpleasantness of an event on NA. Running the model with all moderators simultaneously showed no significant effects. Since the correlations between the moderator variables (see Electronic Supplementary Material 5) were quite large (ie, between 0.034 and 0.658), the moderators were also tested in separate models. This revealed significant effects for illness acceptance (*β* = −0.23, *P* = .016), mindfulness (*β* = −0.24, *P* = .010), and optimism (*β* = −0.28, *P* = .002). LTRs with high levels of illness acceptance, mindfulness, and optimism showed a smaller effect of the unpleasantness of an event on NA.

#### Research aim 3

The third model tested whether the experience of PA protected LTRs from the effect of the unpleasantness of an event on NA. Average high levels of PA (RQ3a) had a significant and negative effect on the association between unpleasantness of an event and NA (= −0.32, *P* = .000). This indicated that the effect of unpleasantness of an event on NA was smaller for LTRs with higher average levels of PA. Momentary PA_(t−1)_ ( ie, levels of PA prior to an unpleasant daily life event; RQ3b) did not significantly protect LTRs from the effect of unpleasantness of an event on NA.

## Discussion

The aim of this study was to identify supportive factors of LTRs daily life resilience. No supportive factors were identified when factors were analyzed in a multivariate model. Exploratory analysis (ie, analyzing supportive factors in separate models) tentatively indicated that LTRs who experienced higher levels of illness acceptance, mindfulness, optimism, and PA in general recovered better from daily life stressors, compared to LTRs with lower levels. We found no effects of tolerance of uncertainty, social support, emotion regulation variability, and momentary PA.

Our findings showed that EMA is an adequate method to study daily life resilience in LTRs. In line with our hypothesis, we found that the more unpleasantly LTRs experienced a daily life event, the more their negative affect increased. Measuring affect in response to a stressor can be seen as an estimation of recovery.^47^ By extension, it can be seen as resilience,^9^ since a spike in NA between 2 consecutive timepoints could be (rapidly) followed by a decrease. A laboratory study confirmed this idea by showing that recovery fifteen minutes after an unpleasant event significantly predicts affective stress reactivity in daily life, but this effect already diminished at 30 minutes after an unpleasant event.^47^ Thus, maintenance of low NA or a small increase of NA could indicate that recovery already occurred between 2 timepoints.^48^ This means that a small or no effect of unpleasantness of events on NA_t_, while controlling for NA_t−1_ indicates a resilient response to a stressor.^12^ Resources or abilities that contribute to a small effect of the association between unpleasantness of events and NA can then be seen as supportive factors of resilience.

In line with other studies, we found that accepting one’s illness helps people to recover from stressful events.^49,50^ Illness acceptance can decrease emotional distress by alleviating feelings of denial, anger, and frustration. It allows individuals to acknowledge their situation and work through their emotions more constructively.^18,19^ A recent scoping review on LTRs highlighted the importance of acceptance of their new reality in order to manage the all-encompassing uncertainty.^15^ Another factor that was found to promote resilience in the current study is mindfulness.^51,52^ Mindfulness helps to reduce stress by promoting relaxation and decreasing the physiological stress response.^14^ Qualitative studies among LTRs showed that being mindful by focusing on the present instead of what might happen in the future prevented LTRs from being overwhelmed by ongoing uncertainty.^53,54^ Optimism was also found to enhance resilience. Being optimistic enhances the ability to find joy despite challenges. Optimistic individuals tend to approach challenges with a problem-solving mindset.^16^ Qualitative studies among LTRs showed that having an optimistic view made it easier to endure their treatment. When they had faith that therapy would work or when they were comparing IT/TT with more invasive treatments such as chemotherapy, they experienced less burden of their side effects of treatment.^4,5^

Contrary to our hypotheses and earlier EMA research, tolerance of uncertainty, social support, and emotion regulation variability were not identified as supportive factors of resilience. In the current study, these factors were measured as static variables, and emotion regulation variability was calculated using a standard deviation-based method. However, the extent to which these factors are expected to be protective stems from how well these factors align with specific contexts and situations.^55,56^ For example, it could upset LTRs when close others want to support them by asking how they feel about the cancer in moments when LTRs are focused on continuing their old life as much as possible.^4^ To unveil how these factors meet patients’ needs in a specific context, it might have been more informative to measure tolerance of uncertainty and social support on a momentary level in response to daily life stressors. Besides, recent research suggests that other methods, for example, using Bray-Curtis dissimilarity, might better capture momentary flexibility in emotion regulation instead of a standard deviation-based method.^57^

We found protective effects of PA on LTRs’ resilience, as those who experienced higher levels of PA in general reported lower increases in NA after an unpleasant event. Following the broaden and build theory, experiencing positive emotions can help in acquiring more resources and lead to increased flexibility.^24^ However, high PA levels immediately before a stressor did not provide a protective effect. This could be the result of a high average PA (M = 74.28), offering little space for heightened PA prior to a stressor to offer an additional contribution to the effect.

### Strengths and limitations

Our study exhibits several strengths. Most notably, we studied resilience as a dynamic process of adaptation (ie, in response to a stressor). While many researchers use this definition, only a handful of studies have examined resilience in response to a stressor.^10^ Most of the existing resilience literature includes self-reported resilience, such as the Brief Resilience Scale or by inquiring about one’s ability to handle today’s challenges at the end of the day.^52^ It is a disadvantage that such questionnaires implicitly assume resilience is a stable trait that does not change over time, across different contexts, or when interacting with various types of adversity.^10^ Currently, the number of studies that examine resilience from a dynamic perspective (ie, in response to a stressor) is increasing.^12^ Another strength is that compliance was noteworthily high for an EMA study (ie, 89%).^58^ Participants expressed a sense of value through their involvement in research. Moreover, they emphasized the importance of contributing to the study, as they missed out on specific psychosocial support and information while obtaining a long-term response to IT/TT.

Our results should also be viewed in light of some limitations. Our sample may not have been representative of the larger group of LTRs’. Since data collection was notably intensive for participants, it is quite possible that patients with mental health challenges were unwilling to participate. The assumption that the individuals within our sample are generally in good mental health is validated by a low average NA, for example, compared to another EMA study in cancer survivors.^59^ Moreover, the relatively low average NA (17.37, with a range of 0 - 100) and low average value of unpleasantness of events (0.44, with a range of 0–3) could indicate the presence of a possible floor effect. That is, when a large proportion of scores on a variable fall close to the minimum score of its scale, data is characterized by skewed distributions and limited variance.^60^ This might have resulted in unaddressed assumption violations. Finally, we did not include a control group in our study. While the present findings are relevant for LTRs, it is unknown whether the findings are specific to this patient group.

### Implications for clinical practice and research

The findings cautiously suggest that promoting resilience may be achievable through cultivating positive psychological factors, such as illness acceptance, optimism, mindfulness, and positive affect. When LTRs experience mental health issues, interventions that aim to increase these positive psychological factors, such as Mindfulness-Based Cognitive Therapy (MBCT) or Acceptance and Commitment Therapy (ACT), might be suitable to increase resilience.^61,62^ MBCT focuses on adopting an accepting stance toward thoughts, emotions, and bodily sensations, helping patients to manage ongoing stressors.^61^ ACT strives to boost psychological flexibility by fostering committed actions that align with personal values,^62^ allowing one to live a value-based life despite illness.

To gain insight into the underlying dynamic interplay of supportive factors and resilience, future research that includes supportive factors at the momentary level is highly recommended.^10^ This provides the opportunity to analyze processes within LTRs and help tailor treatment to LTR’s needs by revealing supportive factors that may benefit from being strengthened further. While studies in cancer patients highlight the importance of goal adjustment, it was not included as a supportive factor in the current study due to the lack of an effective method for assessing the process of goal readjustment.^63,64^ Future research should focus on the impact of letting go of old goals and finding new goals or values on resilience. Finally, studying the role of time since diagnosis in LTRs’ resilience could provide insight into what supports LTRs during different disease phases. The time since diagnosis varied widely among participants in the current study (ie, 8 to 120 months). For example, patients with stage IV melanoma who are in remission for over 5 years are cautiously considered survivors.^65^ These developments could potentially reduce uncertainty and help them to respond more resiliently to everyday life stressors.

### Conclusions

Experiencing recurrent stressors such as medical appointments or response to CT scans and ongoing uncertainty regarding illness and life expectancy can make it difficult for LTRs to cope with daily hassles. Exploratory findings cautiously suggest that a valuable step towards adaptive coping might be helping and empowering LTRs to increase their illness acceptance, mindfulness, optimism, and positive affect. Recognizing and addressing these supportive factors can help LTRs to face daily challenges and enhance their resilience.

## Supplementary Material

kaaf042_suppl_Supplementary_Materials_1-5
