# Supplementary material for: Resilience in advanced cancer patients who obtain a long-term response to immunotherapy or targeted therapy: an Ecological Momentary Assessment study
Source: Ann Behav Med. 2025 Jun 21;59(1):kaaf042. doi: 10.1093/abm/kaaf042 (PMC12204756; doi:10.1093/abm/kaaf042)
Supplement: kaaf042_suppl_Supplementary_Materials_1-5 [file kaaf042_suppl_supplementary_materials_1-5.docx]

**Resilience in advanced cancer patients who obtain a long-term response to immunotherapy or targeted therapy: an Ecological Momentary Assessment study.**

***Electronic Supplemental Materials***

Laura C. Zwanenburg^ab1*^, Eeske van Roekel^cd2^, Karijn P.M. Suijkerbuijk ^e3^, José J. Koldenhof ^e4^ , Olga C. J. Schuurbiers- Siebers^f5^, Janneke van der Stap^g6^, Marije L. van der Lee^ab7,^, Melanie P. J. Schellekens^ab8^

^a^ Tilburg University School of Social and Behavioral Sciences, Center of Research on Psychological disorders and Somatic diseases, Department of Medical and Clinical Psychology, Tilburg.

^b^ Helen Dowling Institute, Centre for Psycho-Oncology, Scientific Research Department, Bilthoven.

^c^ Tilburg University School of Social and Behavioral Sciences, Center of Research on Psychological disorders and Somatic diseases, Department of Developmental Psychology, Tilburg.

^d^ Tilburg University School of Social and Behavioral Sciences, Tilburg Experience Sampling Center, Tilburg.

^e^ University Medical Centre in Utrecht, Department of Medical Oncology, Utrecht University, Utrecht.

^f^ Radboud University Medical Centre, Department of Thoracic Oncology, Nijmegen.

^g^ University Medical Centre in Utrecht, Department of Lung Diseases, Utrecht University, Utrecht.

**S1.** Five Domains of Positive Functioning and promising protective factors of resilience.

| **Domain** | **Promising protective factors included in the current study** |
| --- | --- |
| 1. Attention and Awareness | Mindful awareness |
| 1. Comprehension and Coping | Optimism, Illness acceptance, Tolerance of Uncertainty, Emotion regulation variability |
| 1. Emotions | General level of positive affect (i.e. mean) and level of positive affect before a stressor occurs |
| 1. Goals and Habits | Not included in the current study |
| 1. Virtues and Relationships | Social support |

**S2.** Examples of Personalized feedback.

In the histogram below, your average mood, tiredness, and pain levels during the research period are displayed.


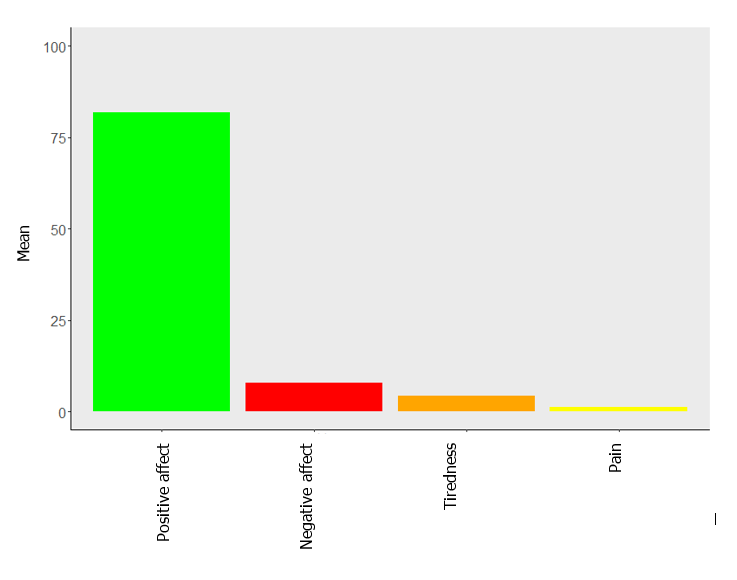


The line chart below is showing the associations of daily events, mood, fatigue, and pain per day. Each chart represents one day. The blue line shows how unpleasant or pleasant an event was and can be read using the right axis. The other lines represent positive and negative mood, fatigue, and pain.

First, look at the blue line to see if a pleasant or unpleasant event occurred. Then, observe whether the other lines remain constant, increase, or decrease compared to the previous time point. For example, the blue line indicates a "slightly pleasant" event, and the green line goes up compared to the previous time point. This might indicate that the pleasant event had a positive impact on the positive mood.


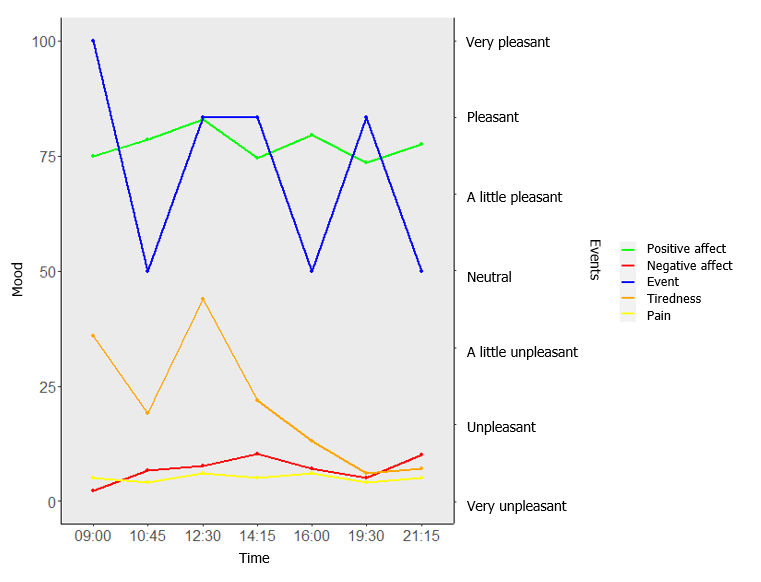


**S3.** Overview specification DSEM models

|  | **Within-person variables** | | **Between-person variables** |
| --- | --- | --- | --- |
| **Model** | **Dependent variable** | **Independent variables** | **Moderators** |
| 1 | NA_(t)_ | NA_(t-1)_, UE_(t)_ | - |
| 2 | NA_(t)_ | NA_(t-1)_, UE_(t)_ | ACC, ToU, MF, SS, OPT, ER var |
| 3a | NA_(t)_ | NA_(t-1)_, UE_(t)_ | PA (mean) |
| 3b | NA_(t)_ | NA_(t-1)_, UE_(t)_, PA_(t-1)_, UE_(t)_ x PA_(t-1)_ | - |

*Note.* NA = negative affect, UE = unpleasantness of event, ACC = illness acceptance, ToU = tolerance of uncertainty, MF = mindfulness, SS = social support, OPT = optimism, ER var = Emotion Regulation variability. UE_(t)_ x PA_(t-1)_ = product variable of unpleasantness of event and positive affect at the previous assessment.

**S4.** Treatment details

| **Treatment** | | **n** | | **%** | |
| --- | --- | --- | --- | --- | --- |
| Immunotherapy | | 39* | | 63.93 | |
| *Durvalumab* | | 2 | |  | |
| *Ipilimumab* | | 6 | |  | |
| *Ipilimumab+Nivolumab* | | 6 | |  | |
| *Nivolumab* | | 11 | |  | |
| *Pembrolizumab* | | 13 | |  | |
| Targeted Therapy | | 22 | | 36.10 | |
| *Afatinib* | | 1 | |  | |
| *Alectinib* | | 4 | |  | |
| *Crizotinib* | | 3 | |  | |
| *Dabrafenib+Trametinib* | | 1 | |  | |
| *Encorafenib+Binimetinib* | | 1 | |  | |
| *Erlotinib* | | 1 | |  | |
| *Lorlatinib* | | 1 | |  | |
| *Osimertinib* | | 9 | |  | |
| *Regorafenib* | | 1 | |  | |

*Type of immunotherapy of one participant is missing

**S5.** Descriptives and between-person correlations between the study variables.

|  | M | SD | Min-Max | Skewness | Kurtosis | 1. | 2. | 3. | 4. | 5. | 6. | 7. |
| --- | --- | --- | --- | --- | --- | --- | --- | --- | --- | --- | --- | --- |
| 1. Negative Affect mean^•^ | 17.37 | 14.16 | 0.53 – 67.95 | 1.21 | 1.59 |  |  |  |  |  |  |  |
| 2. Illness acceptance | 17.03 | 4.06 | 9 – 24 | 0.14 | -0.75 | -.373^**^ |  |  |  |  |  |  |
| 3. Tolerance of uncertainty | 41.04 | 7.39 | 23 – 60 | -0.19 | -0.37 | -.143 | .444^**^ |  |  |  |  |  |
| 4. Mindfulness | 39.82 | 7.12 | 22 – 55 | -0.15 | -0.14 | -.383^**^ | .658^**^ | .348^**^ |  |  |  |  |
| 5. Social support | 5.84 | 1.00 | 3.25 – 7 | -1.13 | 0.37 | -.107 | .162 | .183 | .157 |  |  |  |
| 6. Optimism | 16.32 | 4.84 | 0 – 24 | -0.42 | 0.48 | -.398^**^ | .398^**^ | .257^*^ | .585^**^ | .221 |  |  |
| 7. Emotion regulation variability | 23.14 | 6.82 | 0 – 38.73 | -0.46 | 0.90 | -.274^*^ | .050 | .109 | .034 | .035 | .075 |  |
| 8. Positive affect mean^•^ | 74.28 | 14.15 | 41.33 – 99.34 | 0.03 | -0.66 | -.596^**^ | .603^**^ | .412^**^ | .668^**^ | .276^*^ | .529^**^ | .209 |

*Note*. **Correlation is significant at the 0.01 level (2-tailed). * Correlation is significant at the 0.05 level (2-tailed). Mean^•^ = aggregated mean. Negative Affect mean & Positive Affect mean ranged from 0 to 100.
